# Crystal structure and Hirshfeld surface analysis of *N*-[2-(5-methyl­furan-2-yl)phen­yl]-3-nitro-*N*-[(3-nitro­phen­yl)sulfon­yl]benzene­sulfonamide

**DOI:** 10.1107/S2056989023003523

**Published:** 2023-04-25

**Authors:** Gunay Z. Mammadova, Selbi Annadurdyyeva, Gleb M. Burkin, Victor N. Khrustalev, Mehmet Akkurt, Sema Öztürk Yıldırım, Ajaya Bhattarai

**Affiliations:** a Excellence Center, Baku State University, Z. Xalilov Str. 23, Az 1148 Baku, Azerbaijan; b Peoples’ Friendship University of Russia (RUDN University), 6 Miklukho-Maklaya St., Moscow 117198, Russian Federation; c Zelinsky Institute of Organic Chemistry of RAS, 4, 7 Leninsky Prospect, 119991 Moscow, Russian Federation; d Department of Physics, Faculty of Sciences, Erciyes University, 38039 Kayseri, Türkiye; e Department of Physics, Faculty of Science, Eskisehir Technical University, Yunus Emre Campus, 26470 Eskisehir, Türkiye; f Department of Chemistry, M.M.A.M.C. (Tribhuvan University), Biratnagar, Nepal; University of Neuchâtel, Switzerland

**Keywords:** crystal structure, sulfonamides, hydrogen bonds, π–π stacking inter­actions, Hirshfeld surface analysis

## Abstract

In the title compound, C_23_H_17_N_3_O_9_S_2_, C—H⋯O hydrogen bonds link adjacent mol­ecules in a three-dimensional network, while π–π stacking inter­actions, with centroid–centroid distances of 3.8745 (9) Å, between the furan and an arene ring of one of the two (3-nitro­phen­yl)sulfonyl groups, result in chains parallel to the *a* axis. The Hirshfeld surface analysis indicates that O⋯H/H⋯O (40.1%), H⋯H (27.5%) and C⋯H/H⋯C (12.4%) inter­actions are the most significant contributors to the crystal packing.

## Abstract 2

In the crystal, C—H⋯O hydrogen bonds link adjacent mol­ecules in the three-dimensional network, while π–π stacking inter­actions, with centroid–centroid distances of 3.8745 (9) Å, propagate into chains parallel to the *a* axis.

## 1. Chemical context

**Figure 1:**
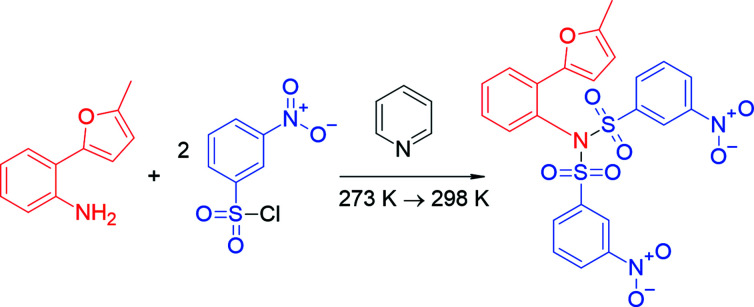
One-pot synthesis of *N*-[2-(5-methyl­furan-2-yl)phen­yl]-3-nitro-*N*-[(3-nitro­phen­yl)sulfon­yl]benzene­sulfonamide.

The synthesis of sulfonamides has been given considerable attention in the literature. A large number of reports are based on various chemical and physical properties, methods of synthesis and application of sulfonamides (Safavora *et al.*, 2019 ▸). The electronic and structural properties of the sulfon­amide moiety make it a bioisostere of such compounds as urea, thio­urea, carbamates and sulfamides (Reitz *et al.*, 2009 ▸; Abdelhamid *et al.*, 2011 ▸; Khalilov *et al.*, 2021 ▸). Linear and cyclic compounds containing sulfonamide fragments have a wide range of biological activity – they possess anti­bacterial properties (Yun *et al.*, 2012 ▸; Nadirova *et al.*, 2021 ▸), show diuretic activity (Logemann *et al.*, 1959 ▸; DeStevens *et al.*, 1959 ▸), are active against seizures (Thiry *et al.*, 2008 ▸) and inhibit various enzymes like human leukocyte elastase and cathepsin G, a HIV-1 protease (Supuran *et al.*, 2003 ▸). Sulfonamides are also used as fungicidal (Chohan *et al.*, 2006 ▸, 2010 ▸) and insecticidal mixtures. The most widely used furan-substituted sul­fonyl­amide is Furosemide, a loop diuretic medication used to treat fluid build-up due to heart failure, kidney disease or liver scarring. Typically, furan-substituted monosulfamides are obtained by treatment of the amines with the corresponding sulfonyl chlorides (Pilipenko *et al.*, 2012 ▸; Butin *et al.*, 2006 ▸; Naghiyev *et al.*, 2020 ▸). It turned out unexpectedly that the inter­action of 2-(α-fur­yl)aniline with sulfochloride containing the electron-withdrawing 3-nitro­phenyl group under the same conditions gives a double sulfaryl­ation product (Fig. 1 ▸), which is possible only with the use of strong bases (Bartsch *et al.*, 1977 ▸; Li *et al.*, 2022 ▸). The obtained product can serve as a compound for studying furan fragment-opening (Pilipenko *et al.*, 2012 ▸; Butin *et al.*, 2006 ▸) or the Diels–Alder reactions of furans (Borisova *et al.*, 2018*a* ▸,*b* ▸; Krishna *et al.*, 2022 ▸; Zubkov *et al.*, 2007 ▸) and for studying biological activity. On the other hand, inter­molecular noncovalent inter­actions organize the mol­ecular aggregates, catalytic inter­mediates, *etc*., which play a critical role in the functional properties of heterocyclic com­pounds (Gurbanov *et al.*, 2020*a* ▸,*b* ▸, 2022 ▸; Ma *et al.*, 2021 ▸; Mahmoudi, *et al.*, 2017*a* ▸,*b* ▸; Mahmudov *et al.*, 2011 ▸, 2022 ▸). 
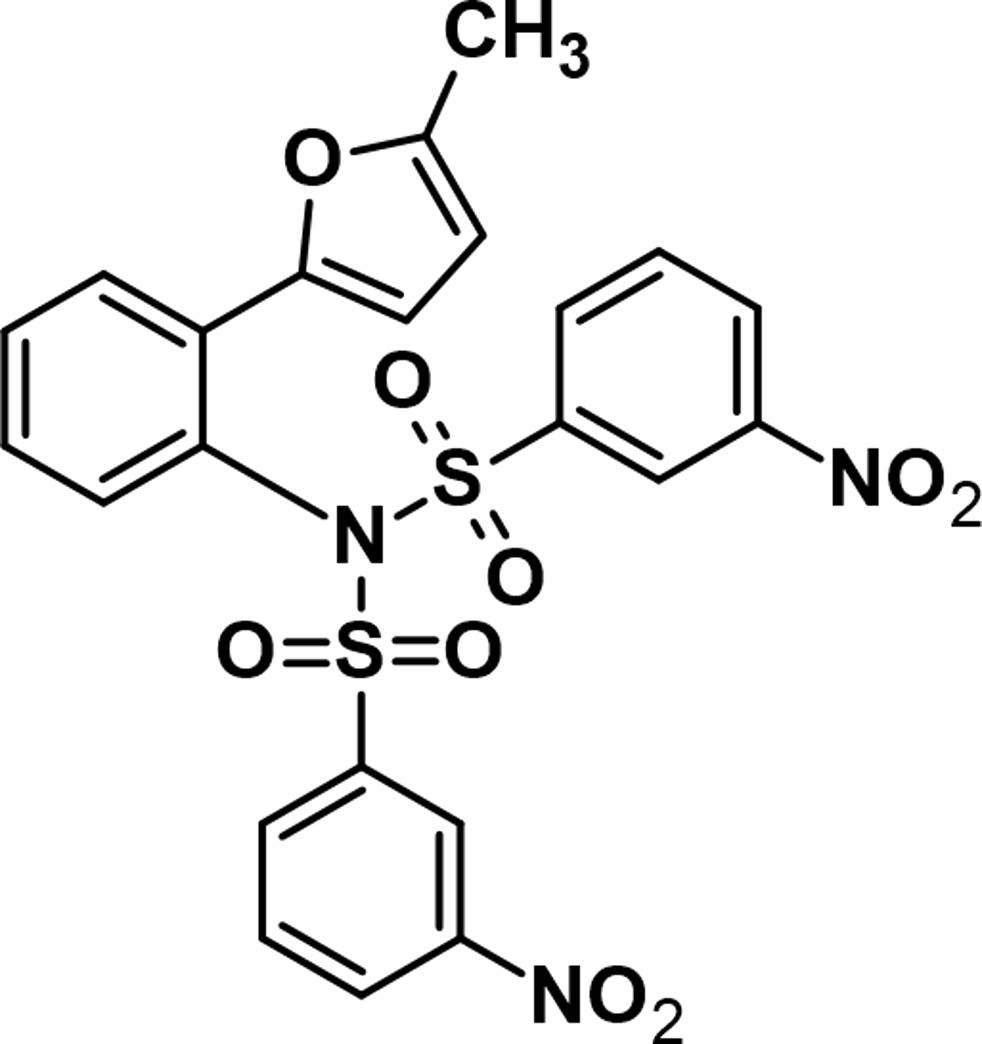


## 2. Structural commentary

**Figure 2:**
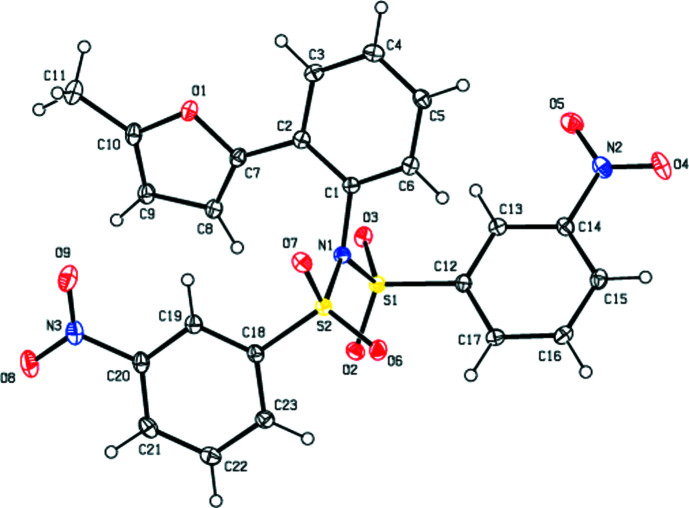
The mol­ecular structure of the title compound, showing the atom labelling and with displacement ellipsoids drawn at the 50% probability level.

In the title compound (Fig. 2 ▸), the angle between the planes of the arene rings (C12–C17 and C18–C23) of the (3-nitro­phen­yl)sulfonyl groups are 40.87 (7)°. The furan ring (O1/C7–C10) is inclined at angles of 51.04 (8) and 12.78 (8)° with respect to the arene rings (C12–C17 and C18–C23) of the (3-nitro­phen­yl)sulfonyl groups, while it makes a dihedral angle of 20.77 (8)° with the plane of the arene ring (C1–C6) attached to the furan ring. The arene ring attached to the furan ring makes dihedral angles of 33.19 (7) and 17.84 (7)°, res­pectively, with the arene rings of the 3-nitro­phen­yl)sulfonyl groups. The geometric properties of the title compound are normal and consistent with those of related compounds listed in the *Database survey* (Section 4).

## 3. Supra­molecular features and Hirshfeld surface analysis

**Figure 3:**
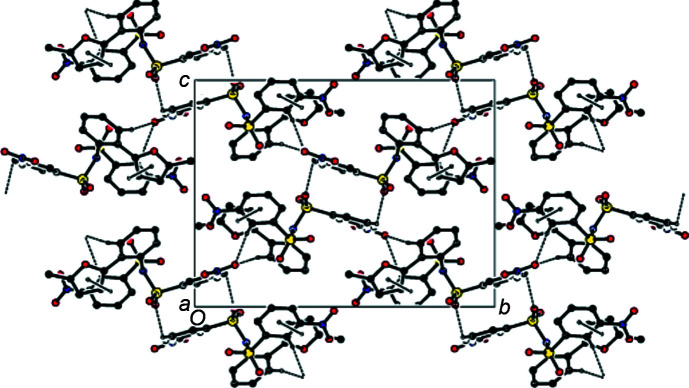
The crystal packing along the *a* axis, showing the C—H⋯O hydrogen-bond network and the π–π stacking inter­actions.

In the crystal of the title compound, mol­ecules are linked by inter­molecular C—H⋯O hydrogen bonds forming the three-dimensional network (Tables 1 ▸ and 2 ▸), while π–π stacking inter­actions {*Cg*1⋯*Cg*4^iv^ = 3.8745 (9) Å [symmetry code: (iv) *x* + 1, *y*, *z*], where *Cg*1 and *Cg*4 are the centroids of the furan ring (atoms O1/C7–C10) and the arene ring (atoms C18–C23) of one of the two (3-nitro­phen­yl)sulfonyl groups; slippage = 1.389 Å} form chains along the *a* axis (Figs. 3 ▸ and 4 ▸).

**Figure 5:**
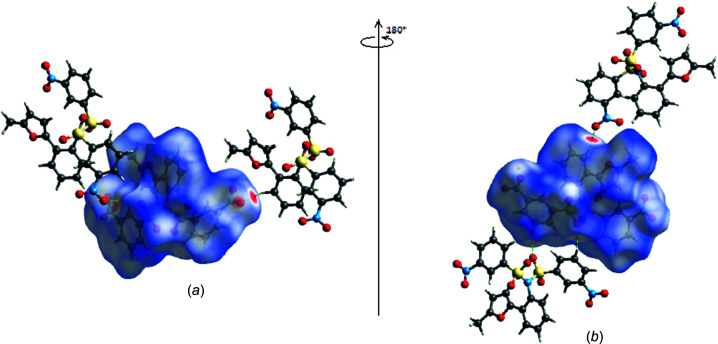
(*a*) Front and (*b*) back views of the three-dimensional Hirshfeld surface, with some inter­molecular C—H⋯O inter­actions shown.

**Figure 6:**
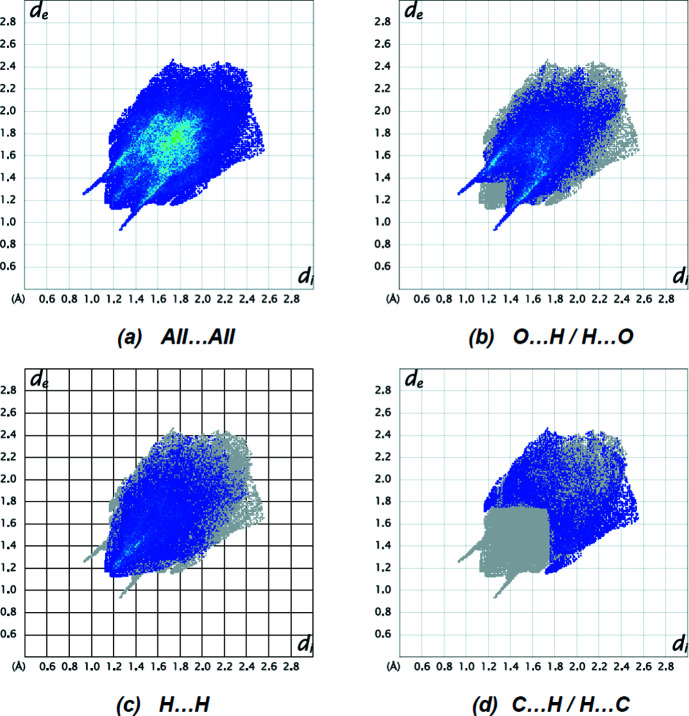
The 2D fingerprint plots for the title mol­ecule, showing (*a*) all inter­actions, and delineated into (*b*) O⋯H/H⋯O, (*c*) H⋯H and (*d*) C⋯H/H⋯C inter­actions. The *d*_i_ and *d*_e_ values are the closest inter­nal and external distances (in Å) from given points on the Hirshfeld surface.

Hirshfeld surfaces were generated for the title mol­ecule using *CrystalExplorer17* (Spackman *et al.*, 2021 ▸). The *d*_norm_ mappings was performed in the range from −0.3170 to +1.1777 a.u. The C—H⋯O inter­actions are indicated by red areas on the Hirshfeld surfaces [Figs. 5 ▸(*a*) and 5(*b*)]. Fingerprint plots (Fig. 6 ▸) reveal that, while O⋯H/H⋯O inter­actions (40.1%) make the largest contributions to the surface contacts (Tables 1 ▸ and 2 ▸), H⋯H (27.5%) and C⋯H/H⋯C (12.4%) contacts are also important. Other less notable linkages are O⋯C/C⋯O (6.0%), O⋯O (5.7%), C⋯C (4.9%), O⋯N/N⋯O (2.0%), N⋯H/H⋯N (1.2%), S⋯C/C⋯S (0.1%) and S⋯O/O⋯S (0.1%).

## 4. Database survey

The nine related compounds found as a result of the search for ‘*N*-(methane­sulfon­yl)-*N*-methyl methane­sulfonamide’ in the Cambridge Structural Database (CSD, Version 5.42, update of September 2021; Groom *et al.*, 2016 ▸) are for *N*-(2-formyl­phen­yl)-4-methyl-*N*-[(4-methyl­phen­yl)sulfon­yl]benzene­sul­fon­amide, *i.e.* CSD refcodes JOBTIF (Kim, 2014 ▸), CEGMIM (Mughal *et al.*, 2012*a* ▸), YAXKAL (Taher & Smith, 2012*a* ▸), OCABUR (Abbassi *et al.*, 2011 ▸), CEGSUE (Mughal *et al.*, 2012*b* ▸), EFASUB (Taher & Smith, 2012*b* ▸), PONZIC (Rizzoli *et al.*, 2009 ▸), AYUPUG (Arshad *et al.*, 2011 ▸) and ROGJON (Li & Song, 2008 ▸).

In JOBTIF (space group *P*2_1_/*n*), mol­ecules are linked by pairs of C—H⋯O hydrogen bonds, forming inversion dimers. In CEGMIM (space group *Pbca*), mol­ecules are connected by C—H⋯O inter­actions into sheets in the *ab* plane. In YAXKAL (space group *P* 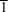 ), mol­ecules associate *via* pairs of N—H⋯N hydrogen bonds, forming a centrosymmetric eight-membered {⋯HNCN}_2_ synthon. The crystal structure of OCABUR (space group *P*2_1_/*c*) is stabilized by inter­molecular C—H⋯O hydrogen bonds. In the crystal of CEGSUE (space group *P* 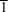 ), the only possible directional inter­actions are very weak C—H⋯π inter­actions and very weak π–π stacking between parallel methyl­phenyl rings. In EFASUB (space group *C*2/*c*), mol­ecules associate *via* N—H⋯N and N—H⋯O hydrogen bonds, forming extended hydrogen-bonded sheets that lie parallel to the *bc* plane. The N—H⋯N hydrogen bonds propagate along the *b*-axis direction, while the N— H⋯O hydrogen bonds propagate along the *c*-axis direction. In the crystal packing of PONZIC (space group *P* 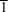 ), mol­ecules are linked into chains parallel to the *a* axis by inter­molecular C—H⋯O hydrogen bonds and π–π stacking inter­actions. In the crystal structure of AYUPUG (space group *P*2_1_/*c*), weak C—H⋯O inter­actions connect the mol­ecules in a zigzag manner along the *a* axis. In ROGJON (space group *Pbca*), the crystal sructure exhibits weak inter­molecular N—H⋯O, C—H⋯O and C—H⋯N hydrogen bonds and π–π inter­actions.

## 5. Synthesis and crystallization

To a solution of 2-(5-methyl­furan-2-yl)aniline (1.09 g, 0.0058 mol) in 7 ml of pyridine under stirring and cooling in an ice-water bath, *m*-nitro­benzene­sulfonyl chloride (2.59 g, 0.0117 mol) was added gradually. The mixture was stirred for 7 h and after completion of the reaction [thin-layer chromatography (TLC) monitoring], the mixture was poured into 90 ml of 6 *M* hydro­chloric acid. The oil which separated was washed with water until it crystallized. The crystals were filtered off, dried and crystallized from an ethanol/di­methyl­formamide (DMF) mixture to give the target disulfonamide as a yellow solid. A single crystal of *N*-[2-(5-methyl­furan-2-yl)phen­yl]-3-nitro-*N*-[(3-nitro­phen­yl)sulfon­yl]benzene­sulfonamide was obtained by slow crystallization from an ethanol/DMF mixture (yield 62%, 1.94 g; m.p. 467–469 K). IR (KBr), ν (cm^−1^): 1176 (ν_s_ SO_2_), 1352 (*br*, ν_as_ SO_2_, ν_s_ NO_2_), 1530 (ν_as_ NO_2_). ^1^H NMR (600.2 MHz, DMSO-*d*_6_) (*J*, Hz): δ 8.60 (*dd*, *J* = 8.1, 1.5 Hz, 2H), 8.36 (*t*, *J* = 1.5 Hz, 2H), 8.25 (*d*, *J* = 8.1 Hz, 2H), 7.94 (*t*, *J* = 8.1 Hz, 2H), 7.75 (*dd*, *J* = 8.1, 1.5 Hz, 1H), 7.59 (*dt*, *J* = 8.1, 1.0 Hz, 1H), 7.38 (*dt*, *J* = 8.1, 1.0 Hz, 1H), 7.12 (*d*, *J* = 8.1 Hz, 1H), 6.60 (*d*, *J* = 3.5 Hz, 1H), 5.81 (*d*, *J* = 3.5 Hz, 1H), 1.86 (*s*, 3H). ^13^C{^1^H} NMR (150.9 MHz, DMSO-*d*_6_): δ 153.2 (2C), 148.2, 147.9, 140.4, 134.9, 133.8, 132.2, 132.1, 132.0, 129.7, 129.0, 128.8, 123.4, 112.0, 108.5, 13.3; MS (ESI) *m*/*z*: [*M* + H]^+^ 544.37. Analysis calculated (%) for C_23_H_17_N_3_O_9_S_2_: C 50.82, H 3.15, N 7.73, S 11.80; found: C 51.07, H 3.17, N 7.56, S 12.03.

## 6. Refinement

Crystal data, data collection and structure refinement details are summarized in Table 3 ▸. All C-bound H atoms were positioned geometrically (C—H = 0.95–0.98 Å) and included as riding contributions with isotropic displacement parameters fixed at 1.2*U*_eq_(C) (1.5 for the methyl groups).

## Supplementary Material

Crystal structure: contains datablock(s) I, global. DOI: 10.1107/S2056989023003523/tx2067sup1.cif

Structure factors: contains datablock(s) I. DOI: 10.1107/S2056989023003523/tx2067Isup2.hkl

Supporting information file. DOI: 10.1107/S2056989023003523/tx2067Isup3.cml

CCDC reference: 2257159

Additional supporting information:  crystallographic information; 3D view; checkCIF report

## Figures and Tables

**Figure 4:**
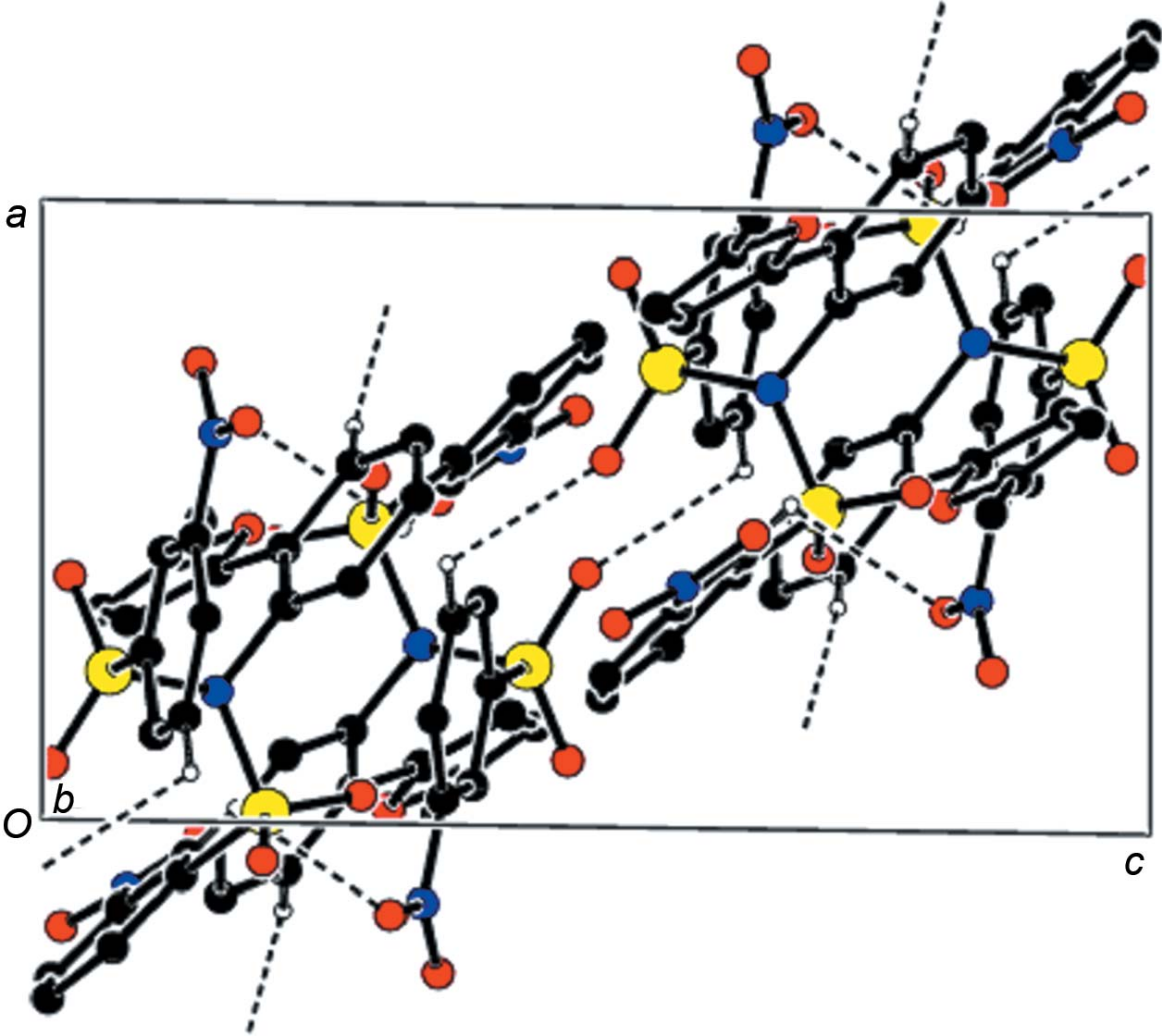
The crystal packing diagram along the *b* axis, showing the inter­molecular C—H⋯O hydrogen bonds.

**Table 1:** Hydrogen-bond geometry (Å, °)

*D*—H⋯*A*	*D*—H	H⋯*A*	*D*⋯*A*	*D*—H⋯*A*
C3—H3⋯O4^i^	0.95	2.31	3.226 (2)	161
C16—H16⋯O2^ii^	0.95	2.53	3.0874 (18)	118
C19—H19⋯O4^iii^	0.95	2.58	2.981 (2)	106

Symmetry codes: (i) 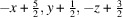 ; (ii) 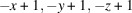 ; (iii) 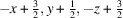 .

**Table 2:** Summary of short inter­atomic contacts (Å).

Contact	Distance	Symmetry operation
N2⋯O3	3.03	−*x* + 2, −*y* + 1, −*z* + 1
H19⋯H15	2.58	−*x* + 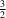 , *y* + 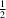 , −*z* + 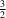
H16⋯O2	2.53	−*x* + 1, −*y* + 1, −*z* + 1
O5⋯H17	2.62	*x* + 1, *y*, *z*
O4⋯H3	2.31	−*x* + 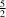 , *y* − 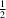 , −*z* + 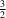
H4⋯H8	2.46	*x* + 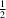 , −*y* + 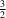 , *z* + 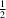
H9⋯O8	2.67	−*x* + 1, −*y* + 2, −*z* + 1

**Table 3:** Experimental details

Crystal data	
Chemical formula	C_23_H_17_N_3_O_9_S_2_
*M*_r_	543.52
Crystal system, space group	Monoclinic, *P*2_1_/*n*
Temperature (K)	100
*a*, *b*, *c* (Å)	8.10683 (6), 19.20010 (15), 14.49754 (10)
β (°)	90.8104 (7)
*V* (Å^3^)	2256.35 (3)
*Z*	4
Radiation type	Cu *K*α
μ (mm^−1^)	2.71
Crystal size (mm)	0.33 × 0.12 × 0.11

Data collection	
Diffractometer	Rigaku XtaLAB Synergy Dualflex HyPix
Absorption correction	Multi-scan (*CrysAlis PRO*; Rigaku OD, 2021 ▸)
*T*_min_, *T*_max_	0.411, 0.725
No. of measured, independent and observed [*I* > 2σ(*I*)] reflections	30531, 4812, 4547
*R*_int_	0.055
(sin θ/λ)_max_ (Å^−1^)	0.634

Refinement	
*R*[*F* ^2^ > 2σ(*F* ^2^)], *wR*(*F* ^2^), *S*	0.036, 0.100, 1.07
No. of reflections	4812
No. of parameters	335
H-atom treatment	H-atom parameters constrained
Δρ_max_, Δρ_min_ (e Å^−3^)	0.54, −0.50

Computer programs: *CrysAlis PRO* (Rigaku OD, 2021 ▸), *SHELXL2016* (Sheldrick, 2015*a* ▸), *SHELXL2016* (Sheldrick, 2015*b* ▸), *ORTEP-3 for Windows* (Farrugia, 2012 ▸) and *PLATON* (Spek, 2020 ▸).

## supplementary crystallographic information

### Crystal data

**Table:**

C_23_H_17_N_3_O_9_S_2_	*F*(000) = 1120
*M**_r_* = 543.52	*D*_x_ = 1.600 Mg m^−^^3^
Monoclinic, *P*2_1_/*n*	Cu *K*α radiation, λ = 1.54184 Å
*a* = 8.10683 (6) Å	Cell parameters from 20996 reflections
*b* = 19.20010 (15) Å	θ = 3.8–77.7°
*c* = 14.49754 (10) Å	µ = 2.71 mm^−^^1^
β = 90.8104 (7)°	*T* = 100 K
*V* = 2256.35 (3) Å^3^	Prismatic needle, yellow
*Z* = 4	0.33 × 0.12 × 0.11 mm

### Data collection

**Table:**

Rigaku XtaLAB Synergy Dualflex HyPix diffractometer	4547 reflections with *I* > 2σ(*I*)
Radiation source: micro-focus sealed X-ray tube	*R*_int_ = 0.055
φ and ω scans	θ_max_ = 77.8°, θ_min_ = 3.8°
Absorption correction: multi-scan (CrysAlis PRO; Rigaku OD, 2021)	*h* = −8→10
*T*_min_ = 0.411, *T*_max_ = 0.725	*k* = −24→24
30531 measured reflections	*l* = −18→18
4812 independent reflections	

### Refinement

**Table:**

Refinement on *F*^2^	Primary atom site location: difference Fourier map
Least-squares matrix: full	Secondary atom site location: difference Fourier map
*R*[*F*^2^ > 2σ(*F*^2^)] = 0.036	Hydrogen site location: inferred from neighbouring sites
*wR*(*F*^2^) = 0.100	H-atom parameters constrained
*S* = 1.07	*w* = 1/[σ^2^(*F*_o_^2^) + (0.0588*P*)^2^ + 0.9267*P*] where *P* = (*F*_o_^2^ + 2*F*_c_^2^)/3
4812 reflections	(Δ/σ)_max_ = 0.001
335 parameters	Δρ_max_ = 0.54 e Å^−^^3^
0 restraints	Δρ_min_ = −0.50 e Å^−^^3^

### Special details

**Table:**

Geometry. All esds (except the esd in the dihedral angle between two l.s. planes) are estimated using the full covariance matrix. The cell esds are taken into account individually in the estimation of esds in distances, angles and torsion angles; correlations between esds in cell parameters are only used when they are defined by crystal symmetry. An approximate (isotropic) treatment of cell esds is used for estimating esds involving l.s. planes.

### Fractional atomic coordinates and isotropic or equivalent isotropic displacement parameters (Å^2^)

**Table:**

	*x*	*y*	*z*	*U*_iso_*/*U*_eq_	
S1	0.74119 (4)	0.62643 (2)	0.56167 (2)	0.01729 (11)	
S2	0.51739 (4)	0.68042 (2)	0.70267 (2)	0.01806 (11)	
O1	0.97431 (14)	0.86445 (5)	0.68665 (7)	0.0223 (2)	
O2	0.59124 (13)	0.63170 (6)	0.50939 (7)	0.0225 (2)	
O3	0.89353 (13)	0.65098 (5)	0.52573 (7)	0.0220 (2)	
O4	1.14629 (16)	0.36623 (7)	0.68499 (9)	0.0341 (3)	
O5	1.23988 (15)	0.46683 (7)	0.64260 (9)	0.0340 (3)	
O6	0.43825 (13)	0.61417 (6)	0.70008 (8)	0.0241 (2)	
O7	0.54611 (13)	0.71571 (6)	0.78814 (7)	0.0239 (2)	
O8	0.32788 (17)	0.96375 (7)	0.51794 (10)	0.0377 (3)	
O9	0.47463 (18)	0.94819 (6)	0.64251 (9)	0.0357 (3)	
N1	0.71051 (15)	0.67238 (6)	0.65814 (8)	0.0179 (2)	
N2	1.12743 (17)	0.42581 (7)	0.65689 (9)	0.0247 (3)	
N3	0.38951 (18)	0.92648 (7)	0.57790 (10)	0.0278 (3)	
C1	0.85072 (17)	0.68131 (7)	0.72031 (10)	0.0175 (3)	
C2	0.94303 (17)	0.74318 (7)	0.71871 (10)	0.0186 (3)	
C3	1.07789 (18)	0.74767 (8)	0.78022 (10)	0.0220 (3)	
H3	1.1415	0.7892	0.7821	0.026*	
C4	1.12052 (19)	0.69311 (9)	0.83828 (11)	0.0239 (3)	
H4	1.2142	0.6972	0.8780	0.029*	
C5	1.02746 (19)	0.63238 (8)	0.83891 (10)	0.0224 (3)	
H5	1.0569	0.5949	0.8787	0.027*	
C6	0.89046 (18)	0.62723 (7)	0.78039 (10)	0.0200 (3)	
H6	0.8238	0.5866	0.7815	0.024*	
C7	0.90565 (18)	0.80227 (8)	0.65835 (10)	0.0199 (3)	
C8	0.82198 (19)	0.81274 (8)	0.57740 (11)	0.0224 (3)	
H8	0.7638	0.7786	0.5424	0.027*	
C9	0.8383 (2)	0.88476 (8)	0.55513 (11)	0.0242 (3)	
H9	0.7929	0.9077	0.5025	0.029*	
C10	0.9303 (2)	0.91413 (8)	0.62300 (11)	0.0238 (3)	
C11	0.9849 (2)	0.98641 (8)	0.64350 (12)	0.0303 (4)	
H11A	0.9245	1.0043	0.6966	0.045*	
H11B	0.9628	1.0161	0.5897	0.045*	
H11C	1.1034	0.9866	0.6577	0.045*	
C12	0.77285 (17)	0.53925 (7)	0.59476 (10)	0.0183 (3)	
C13	0.93448 (18)	0.51767 (8)	0.61163 (10)	0.0193 (3)	
H13	1.0250	0.5487	0.6053	0.023*	
C14	0.95731 (18)	0.44913 (8)	0.63798 (10)	0.0201 (3)	
C15	0.8286 (2)	0.40242 (8)	0.64729 (10)	0.0227 (3)	
H15	0.8491	0.3557	0.6658	0.027*	
C16	0.66862 (19)	0.42513 (8)	0.62896 (10)	0.0228 (3)	
H16	0.5788	0.3937	0.6342	0.027*	
C17	0.63988 (18)	0.49387 (8)	0.60302 (10)	0.0203 (3)	
H17	0.5306	0.5097	0.5911	0.024*	
C18	0.41201 (17)	0.73702 (7)	0.62594 (10)	0.0190 (3)	
C19	0.44463 (18)	0.80786 (8)	0.63208 (10)	0.0203 (3)	
H19	0.5225	0.8258	0.6755	0.024*	
C20	0.35845 (18)	0.85112 (8)	0.57207 (11)	0.0219 (3)	
C21	0.24370 (19)	0.82640 (9)	0.50838 (11)	0.0248 (3)	
H21	0.1887	0.8575	0.4672	0.030*	
C22	0.21043 (19)	0.75567 (9)	0.50568 (11)	0.0254 (3)	
H22	0.1298	0.7381	0.4635	0.030*	
C23	0.29462 (18)	0.71020 (8)	0.56445 (11)	0.0221 (3)	
H23	0.2724	0.6616	0.5627	0.027*	

### Atomic displacement parameters (Å^2^)

**Table:**

	*U*^11^	*U*^22^	*U*^33^	*U*^12^	*U*^13^	*U*^23^
S1	0.01830 (18)	0.01684 (18)	0.01676 (18)	0.00090 (11)	0.00114 (13)	−0.00100 (11)
S2	0.01788 (18)	0.01795 (18)	0.01841 (18)	0.00142 (12)	0.00264 (12)	0.00124 (12)
O1	0.0280 (5)	0.0168 (5)	0.0222 (5)	−0.0019 (4)	0.0030 (4)	0.0000 (4)
O2	0.0224 (5)	0.0235 (5)	0.0213 (5)	0.0036 (4)	−0.0038 (4)	−0.0022 (4)
O3	0.0243 (5)	0.0196 (5)	0.0222 (5)	−0.0006 (4)	0.0055 (4)	0.0000 (4)
O4	0.0361 (7)	0.0335 (6)	0.0329 (6)	0.0170 (5)	0.0079 (5)	0.0117 (5)
O5	0.0209 (6)	0.0362 (7)	0.0449 (7)	0.0040 (5)	0.0013 (5)	0.0015 (5)
O6	0.0231 (5)	0.0197 (5)	0.0296 (6)	−0.0012 (4)	0.0038 (4)	0.0043 (4)
O7	0.0242 (5)	0.0280 (6)	0.0195 (5)	0.0049 (4)	0.0022 (4)	−0.0011 (4)
O8	0.0431 (7)	0.0264 (6)	0.0436 (7)	0.0081 (5)	0.0027 (6)	0.0128 (5)
O9	0.0519 (8)	0.0216 (6)	0.0337 (7)	−0.0010 (5)	0.0018 (6)	−0.0020 (5)
N1	0.0170 (6)	0.0182 (6)	0.0184 (6)	0.0010 (4)	0.0010 (4)	−0.0020 (4)
N2	0.0251 (7)	0.0284 (7)	0.0207 (6)	0.0082 (5)	0.0039 (5)	−0.0002 (5)
N3	0.0324 (7)	0.0216 (7)	0.0297 (7)	0.0050 (5)	0.0085 (6)	0.0032 (5)
C1	0.0161 (6)	0.0195 (7)	0.0169 (6)	0.0005 (5)	0.0016 (5)	−0.0019 (5)
C2	0.0188 (6)	0.0190 (7)	0.0181 (6)	0.0002 (5)	0.0042 (5)	−0.0008 (5)
C3	0.0206 (7)	0.0234 (7)	0.0220 (7)	−0.0033 (6)	0.0016 (5)	−0.0023 (6)
C4	0.0205 (7)	0.0304 (8)	0.0207 (7)	−0.0003 (6)	−0.0009 (5)	−0.0005 (6)
C5	0.0243 (7)	0.0245 (7)	0.0184 (7)	0.0035 (6)	−0.0002 (6)	0.0022 (5)
C6	0.0217 (7)	0.0189 (7)	0.0194 (7)	−0.0006 (5)	0.0036 (5)	−0.0003 (5)
C7	0.0205 (7)	0.0172 (6)	0.0221 (7)	−0.0010 (5)	0.0049 (5)	−0.0022 (5)
C8	0.0243 (7)	0.0195 (7)	0.0235 (7)	−0.0012 (5)	0.0011 (6)	0.0016 (5)
C9	0.0265 (7)	0.0206 (7)	0.0257 (8)	0.0007 (6)	0.0033 (6)	0.0041 (6)
C10	0.0276 (7)	0.0185 (7)	0.0257 (7)	0.0014 (6)	0.0083 (6)	0.0022 (6)
C11	0.0398 (9)	0.0199 (7)	0.0315 (8)	−0.0020 (6)	0.0061 (7)	−0.0004 (6)
C12	0.0197 (7)	0.0173 (6)	0.0179 (6)	0.0013 (5)	0.0017 (5)	−0.0019 (5)
C13	0.0186 (7)	0.0210 (7)	0.0182 (6)	−0.0007 (5)	0.0023 (5)	−0.0019 (5)
C14	0.0209 (7)	0.0221 (7)	0.0172 (6)	0.0044 (5)	0.0014 (5)	−0.0021 (5)
C15	0.0312 (8)	0.0187 (7)	0.0181 (7)	0.0004 (6)	0.0009 (6)	−0.0011 (5)
C16	0.0266 (7)	0.0218 (7)	0.0199 (7)	−0.0060 (6)	0.0001 (6)	−0.0011 (5)
C17	0.0193 (7)	0.0225 (7)	0.0190 (7)	−0.0018 (5)	0.0001 (5)	−0.0020 (5)
C18	0.0167 (6)	0.0197 (7)	0.0207 (7)	0.0030 (5)	0.0040 (5)	0.0005 (5)
C19	0.0205 (7)	0.0202 (7)	0.0202 (7)	0.0021 (5)	0.0042 (5)	−0.0009 (5)
C20	0.0229 (7)	0.0187 (7)	0.0243 (7)	0.0039 (5)	0.0072 (6)	0.0019 (6)
C21	0.0217 (7)	0.0293 (8)	0.0235 (7)	0.0071 (6)	0.0040 (6)	0.0048 (6)
C22	0.0199 (7)	0.0312 (8)	0.0250 (7)	0.0024 (6)	−0.0006 (6)	−0.0011 (6)
C23	0.0182 (7)	0.0237 (7)	0.0245 (7)	0.0005 (5)	0.0021 (5)	−0.0014 (6)

### Geometric parameters (Å, º)

**Table:**

S1—O2	1.4269 (11)	C8—C9	1.427 (2)
S1—O3	1.4274 (11)	C8—H8	0.9500
S1—N1	1.6752 (12)	C9—C10	1.350 (2)
S1—C12	1.7591 (15)	C9—H9	0.9500
S2—O6	1.4249 (11)	C10—C11	1.485 (2)
S2—O7	1.4283 (11)	C11—H11A	0.9800
S2—N1	1.7089 (12)	C11—H11B	0.9800
S2—C18	1.7667 (15)	C11—H11C	0.9800
O1—C10	1.3709 (19)	C12—C17	1.393 (2)
O1—C7	1.3773 (18)	C12—C13	1.393 (2)
O4—N2	1.2231 (18)	C13—C14	1.382 (2)
O5—N2	1.2247 (19)	C13—H13	0.9500
O8—N3	1.227 (2)	C14—C15	1.384 (2)
O9—N3	1.228 (2)	C15—C16	1.390 (2)
N1—C1	1.4507 (18)	C15—H15	0.9500
N2—C14	1.4720 (19)	C16—C17	1.391 (2)
N3—C20	1.471 (2)	C16—H16	0.9500
C1—C6	1.390 (2)	C17—H17	0.9500
C1—C2	1.404 (2)	C18—C19	1.388 (2)
C2—C3	1.404 (2)	C18—C23	1.393 (2)
C2—C7	1.462 (2)	C19—C20	1.385 (2)
C3—C4	1.385 (2)	C19—H19	0.9500
C3—H3	0.9500	C20—C21	1.385 (2)
C4—C5	1.389 (2)	C21—C22	1.385 (2)
C4—H4	0.9500	C21—H21	0.9500
C5—C6	1.392 (2)	C22—C23	1.392 (2)
C5—H5	0.9500	C22—H22	0.9500
C6—H6	0.9500	C23—H23	0.9500
C7—C8	1.362 (2)		
			
O2—S1—O3	121.18 (7)	C10—C9—H9	126.5
O2—S1—N1	105.71 (6)	C8—C9—H9	126.5
O3—S1—N1	105.68 (6)	C9—C10—O1	109.63 (13)
O2—S1—C12	109.40 (7)	C9—C10—C11	134.12 (15)
O3—S1—C12	106.87 (6)	O1—C10—C11	116.20 (14)
N1—S1—C12	107.24 (6)	C10—C11—H11A	109.5
O6—S2—O7	120.94 (7)	C10—C11—H11B	109.5
O6—S2—N1	108.91 (6)	H11A—C11—H11B	109.5
O7—S2—N1	103.39 (6)	C10—C11—H11C	109.5
O6—S2—C18	108.60 (7)	H11A—C11—H11C	109.5
O7—S2—C18	109.02 (7)	H11B—C11—H11C	109.5
N1—S2—C18	104.78 (6)	C17—C12—C13	121.75 (13)
C10—O1—C7	107.60 (12)	C17—C12—S1	120.58 (11)
C1—N1—S1	117.17 (9)	C13—C12—S1	117.67 (11)
C1—N1—S2	117.94 (9)	C14—C13—C12	116.99 (13)
S1—N1—S2	120.70 (7)	C14—C13—H13	121.5
O4—N2—O5	124.61 (14)	C12—C13—H13	121.5
O4—N2—C14	117.32 (13)	C13—C14—C15	123.07 (14)
O5—N2—C14	118.07 (13)	C13—C14—N2	117.55 (13)
O8—N3—O9	124.14 (15)	C15—C14—N2	119.37 (13)
O8—N3—C20	117.73 (15)	C14—C15—C16	118.73 (14)
O9—N3—C20	118.13 (13)	C14—C15—H15	120.6
C6—C1—C2	121.55 (13)	C16—C15—H15	120.6
C6—C1—N1	118.27 (12)	C15—C16—C17	120.10 (14)
C2—C1—N1	120.18 (12)	C15—C16—H16	119.9
C3—C2—C1	116.87 (13)	C17—C16—H16	119.9
C3—C2—C7	119.05 (13)	C16—C17—C12	119.34 (14)
C1—C2—C7	124.08 (13)	C16—C17—H17	120.3
C4—C3—C2	121.68 (14)	C12—C17—H17	120.3
C4—C3—H3	119.2	C19—C18—C23	122.06 (14)
C2—C3—H3	119.2	C19—C18—S2	118.18 (12)
C3—C4—C5	120.55 (14)	C23—C18—S2	119.69 (11)
C3—C4—H4	119.7	C20—C19—C18	116.97 (14)
C5—C4—H4	119.7	C20—C19—H19	121.5
C4—C5—C6	118.98 (14)	C18—C19—H19	121.5
C4—C5—H5	120.5	C19—C20—C21	122.77 (14)
C6—C5—H5	120.5	C19—C20—N3	118.00 (14)
C1—C6—C5	120.32 (13)	C21—C20—N3	119.22 (14)
C1—C6—H6	119.8	C20—C21—C22	118.91 (15)
C5—C6—H6	119.8	C20—C21—H21	120.5
C8—C7—O1	108.84 (13)	C22—C21—H21	120.5
C8—C7—C2	136.62 (14)	C21—C22—C23	120.27 (15)
O1—C7—C2	114.51 (13)	C21—C22—H22	119.9
C7—C8—C9	106.95 (14)	C23—C22—H22	119.9
C7—C8—H8	126.5	C22—C23—C18	118.97 (14)
C9—C8—H8	126.5	C22—C23—H23	120.5
C10—C9—C8	106.96 (14)	C18—C23—H23	120.5
			
O2—S1—N1—C1	−174.19 (10)	O2—S1—C12—C17	−25.39 (14)
O3—S1—N1—C1	−44.58 (11)	O3—S1—C12—C17	−158.26 (11)
C12—S1—N1—C1	69.16 (11)	N1—S1—C12—C17	88.80 (13)
O2—S1—N1—S2	29.26 (10)	O2—S1—C12—C13	154.26 (11)
O3—S1—N1—S2	158.87 (8)	O3—S1—C12—C13	21.39 (13)
C12—S1—N1—S2	−87.39 (9)	N1—S1—C12—C13	−91.55 (12)
O6—S2—N1—C1	−112.41 (11)	C17—C12—C13—C14	−0.6 (2)
O7—S2—N1—C1	17.40 (12)	S1—C12—C13—C14	179.70 (10)
C18—S2—N1—C1	131.55 (11)	C12—C13—C14—C15	0.4 (2)
O6—S2—N1—S1	43.96 (10)	C12—C13—C14—N2	−179.44 (12)
O7—S2—N1—S1	173.77 (8)	O4—N2—C14—C13	176.14 (13)
C18—S2—N1—S1	−72.08 (9)	O5—N2—C14—C13	−4.6 (2)
S1—N1—C1—C6	−81.02 (15)	O4—N2—C14—C15	−3.7 (2)
S2—N1—C1—C6	76.19 (15)	O5—N2—C14—C15	175.51 (14)
S1—N1—C1—C2	98.73 (14)	C13—C14—C15—C16	0.3 (2)
S2—N1—C1—C2	−104.06 (13)	N2—C14—C15—C16	−179.83 (13)
C6—C1—C2—C3	0.4 (2)	C14—C15—C16—C17	−0.9 (2)
N1—C1—C2—C3	−179.36 (12)	C15—C16—C17—C12	0.7 (2)
C6—C1—C2—C7	−178.88 (13)	C13—C12—C17—C16	0.1 (2)
N1—C1—C2—C7	1.4 (2)	S1—C12—C17—C16	179.75 (11)
C1—C2—C3—C4	1.5 (2)	O6—S2—C18—C19	166.10 (11)
C7—C2—C3—C4	−179.21 (14)	O7—S2—C18—C19	32.49 (13)
C2—C3—C4—C5	−1.6 (2)	N1—S2—C18—C19	−77.64 (12)
C3—C4—C5—C6	−0.1 (2)	O6—S2—C18—C23	−11.09 (14)
C2—C1—C6—C5	−2.1 (2)	O7—S2—C18—C23	−144.70 (12)
N1—C1—C6—C5	177.64 (13)	N1—S2—C18—C23	105.16 (12)
C4—C5—C6—C1	1.9 (2)	C23—C18—C19—C20	−1.9 (2)
C10—O1—C7—C8	0.89 (16)	S2—C18—C19—C20	−179.03 (10)
C10—O1—C7—C2	179.24 (12)	C18—C19—C20—C21	0.3 (2)
C3—C2—C7—C8	158.66 (17)	C18—C19—C20—N3	179.36 (12)
C1—C2—C7—C8	−22.1 (3)	O8—N3—C20—C19	171.70 (14)
C3—C2—C7—O1	−19.06 (19)	O9—N3—C20—C19	−8.6 (2)
C1—C2—C7—O1	160.19 (13)	O8—N3—C20—C21	−9.2 (2)
O1—C7—C8—C9	−0.59 (17)	O9—N3—C20—C21	170.48 (14)
C2—C7—C8—C9	−178.40 (16)	C19—C20—C21—C22	1.5 (2)
C7—C8—C9—C10	0.06 (18)	N3—C20—C21—C22	−177.54 (13)
C8—C9—C10—O1	0.49 (17)	C20—C21—C22—C23	−1.8 (2)
C8—C9—C10—C11	−176.60 (17)	C21—C22—C23—C18	0.2 (2)
C7—O1—C10—C9	−0.86 (16)	C19—C18—C23—C22	1.7 (2)
C7—O1—C10—C11	176.82 (13)	S2—C18—C23—C22	178.77 (11)

### Hydrogen-bond geometry (Å, º)

**Table:**

*D*—H···*A*	*D*—H	H···*A*	*D*···*A*	*D*—H···*A*
C3—H3···O1	0.95	2.41	2.7460 (18)	101
C3—H3···O4^i^	0.95	2.31	3.226 (2)	161
C13—H13···O3	0.95	2.51	2.8637 (18)	102
C16—H16···O2^ii^	0.95	2.53	3.0874 (18)	118
C19—H19···O4^iii^	0.95	2.58	2.981 (2)	106
C23—H23···O6	0.95	2.56	2.9252 (19)	103

Symmetry codes: (i) −*x*+5/2, *y*+1/2, −*z*+3/2; (ii) −*x*+1, −*y*+1, −*z*+1; (iii) −*x*+3/2, *y*+1/2, −*z*+3/2.
